# Supplementary material for: Alkylresorcinols as New Modulators of the Metabolic Activity of the Gut Microbiota
Source: Int J Mol Sci. 2023 Sep 18;24(18):14206. doi: 10.3390/ijms241814206 (PMC10532030; doi:10.3390/ijms241814206)
Supplement: Supplementary file 1 [file ijms-24-14206-s001.zip › ijms-2539051-supplementary/Supplementary Tables.zip!/Supplementary Tables/Supplementary Table 1_IJMS.pdf]

Supplementary Table S1. Primers used to amplify the V3-V4 region of bacterial 16S rRNA gene.

Forward Primer:	**TCGTCGGCAGCGTCAGATGTGTATAAGAGACAG**CCTACGGGAGGCAGCAG
Reverse Primer:	**GTCTCGTGGGCTCGGAGATGTGTATAAGAGACAG**GACTACAAGGGTATCTAATCC
