# Supplementary material for: Alkylresorcinols as New Modulators of the Metabolic Activity of the Gut Microbiota
Source: Int J Mol Sci. 2023 Sep 18;24(18):14206. doi: 10.3390/ijms241814206 (PMC10532030; doi:10.3390/ijms241814206)
Supplement: Supplementary file 1 [file ijms-24-14206-s001.zip › ijms-2539051-supplementary/Supplementary Tables.zip!/Supplementary Tables/Supplementary Table 2_IJMS.pdf]

Supplementary Table S2. Characteristics of mouse gut microbiota metagenome sequencing analysis. Mean values and standard deviation (SD) for every group are shown.

BL - C57BL/6 mice

DB – db/db mice

LDLR – Ldlr(-/-) mice

RCD – row chow diet

HFD – high fat diet

C5 – olivetol supplementation to a diet

Animal group	BL/RCD			BL/HFD		
Feature	Mean	SD	N of samples	Mean	SD	N of samples
forward sequence count	120068,5	30077,18654	10	108843,1	17064,21	10
reverse sequence count	120068,5	30077,18654	10	108843,1	17064,21	10
Taxa_S	1663,9	177,7941694	10	1296,7	123,0357	10
Animal group	BL/RCD+C5			BL/HFD+C5		
Feature	Mean	SD	N of samples	Mean	SD	N of samples
forward sequence count	108320,9	8117,759735	9	99259,2	17672,14	10
reverse sequence count	108320,9	8117,759735	9	99259,2	17672,14	10
Taxa_S	1494,111	169,6875396	9	1283,4	84,03994	10
Animal group	DB/RCD			DB/RCD+C5		
Feature	Mean	SD	N of samples	Mean	SD	N of samples
forward sequence count	116371	8139,8291	10	124596,5	31921,57	9
reverse sequence count	116371	8139,8291	10	124596,5	31921,57	9
Taxa_S	1141,8	112,6427983	10	1232,222	243,759	9
Animal group	LDLR/RCD			LDLR/RCD+C5		
Feature	Mean	SD	N of samples	Mean	SD	N of samples
forward sequence count	143189	20253,58531	10	102636,7	10937,48	10
reverse sequence count	143189	20253,58531	10	102636,7	10937,48	10
Taxa_S	1042,4	140,3782193	10	703,9	141,4838	10
